# Supplementary material for: FUS regulates a subset of snoRNA expression and modulates the level of rRNA modifications
Source: Sci Rep. 2023 Feb 20;13:2974. doi: 10.1038/s41598-023-30068-2 (PMC9941101; doi:10.1038/s41598-023-30068-2)
Supplement: Supplementary file 1 — Supplementary Information 1. [file 41598_2023_30068_MOESM1_ESM.docx]

**Supplementary Information for**

**Manuscript title**

**FUS regulates a subset of snoRNA expression and modulates the level of rRNA modifications**

**Authors**

Kishor Gawade^1,2^, Patrycja Plewka^1,2^, Sophia J Häfner^3^, Anders H Lund^3^, Virginie Marchand^4^, Yuri Motorin^4^, Michal W Szczesniak^5^, Katarzyna D Raczynska^1,2*^

* To whom correspondence should be addressed. Department of Gene Expression, Uniwersytetu Poznanskiego 10, 61-614 Poznan, Poland, tel: +48618291901; fax: (+4861)8295636; email: doracz@amu.edu.pl

**This PDF file includes:**

Supplementary Figures S1 to S9

Supplementary Tables S3 and S5


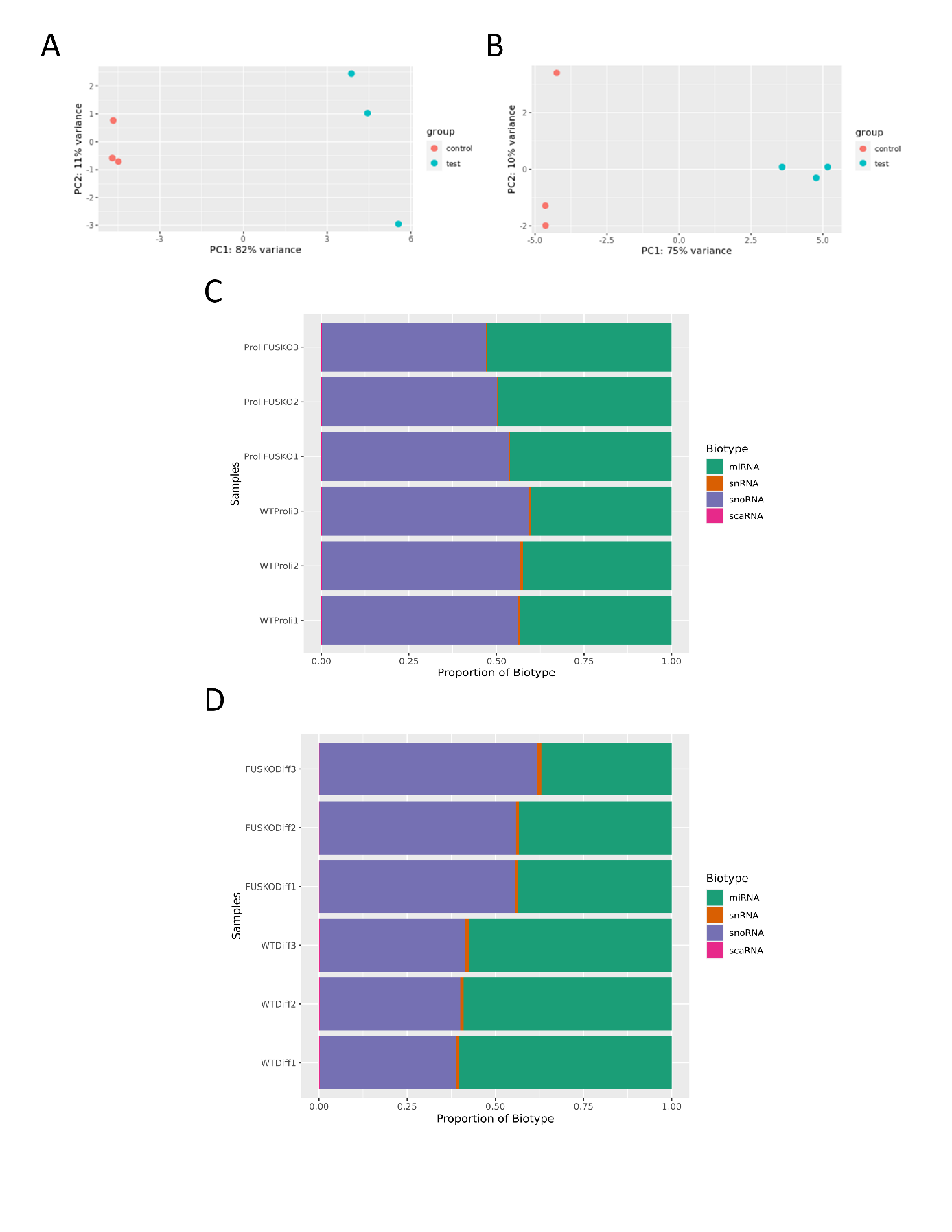


**Supplementary Fig. S1**. PCA (principal component analysis) plots generated using ggplot2 library in R. Raw count matrix generated from FeatureCounts was used with rlog (regularized log) transformation in DESeq2 R package. Control = wild-type cells, test = FUS KO cells (A and B). Proportion of small RNA biotypes counted by the FeatureCounts tool and plotted using libraries ggplot2 and reshape2 in R. X-axis denotes proportion of each biotype in the samples and on Y-axis are the sample names with three biological replicates each (C and D).


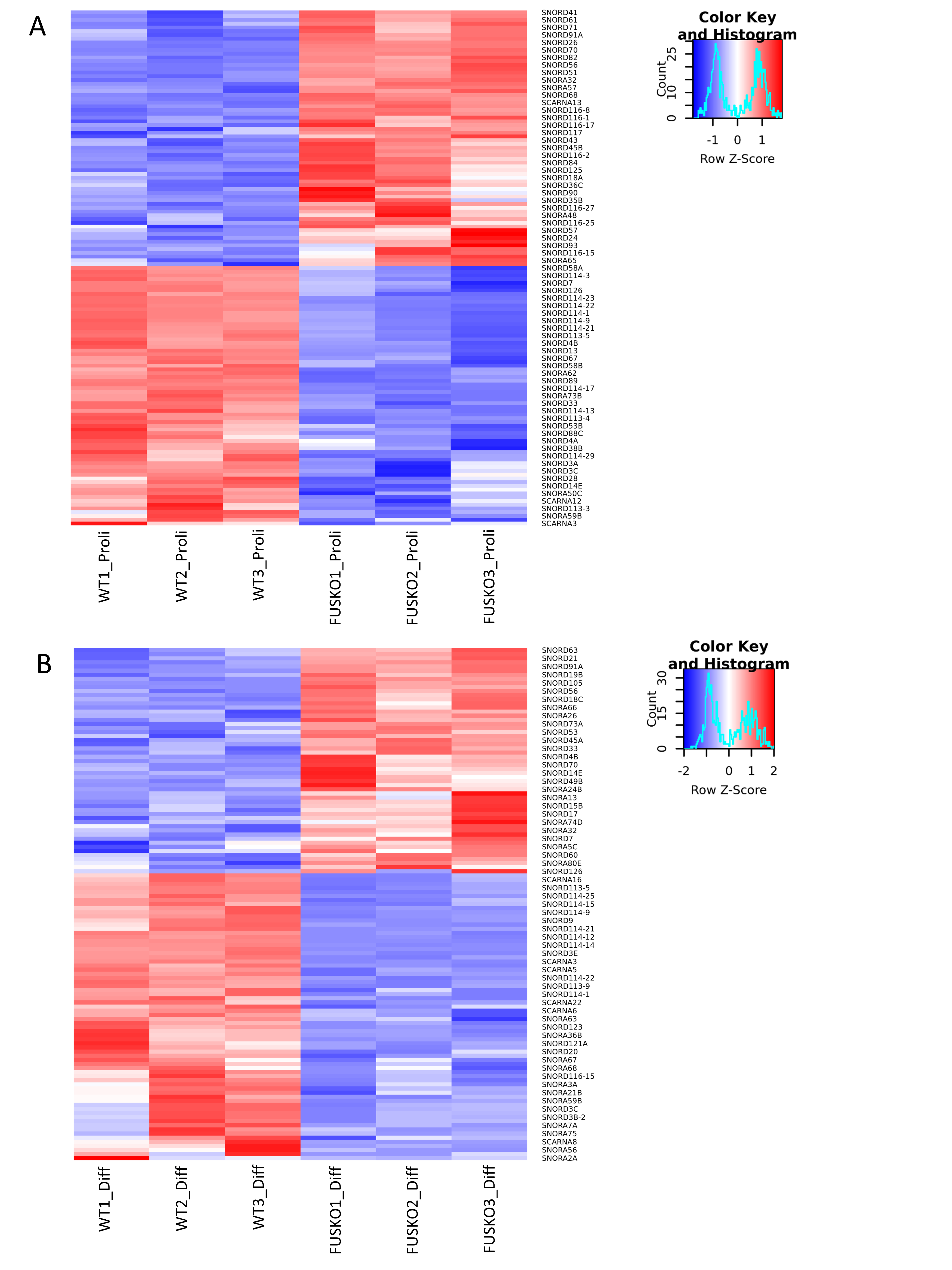


**Supplementary Fig. S2.** Differentially expressed snoRNAs in the proliferating (Proli) (A) and differentiated (Diff) (B) SH-SY5Y WT and FUS KO cells identified by small RNA sequencing (listed in Supplementary Table S1). For clarity, in these heatmaps only selected rows are depicted by snoRNA names. DESeq normalized read count was used to generate heatmaps for differentially expressed genes with Heatmap.2 function of gplots 3.1.3 R package (https://CRAN.R-project.org/package=gplots). Padj (p-adjusted value cut off ≤0.05). The blue and red colors show a low and high level of expression, respectively.

**
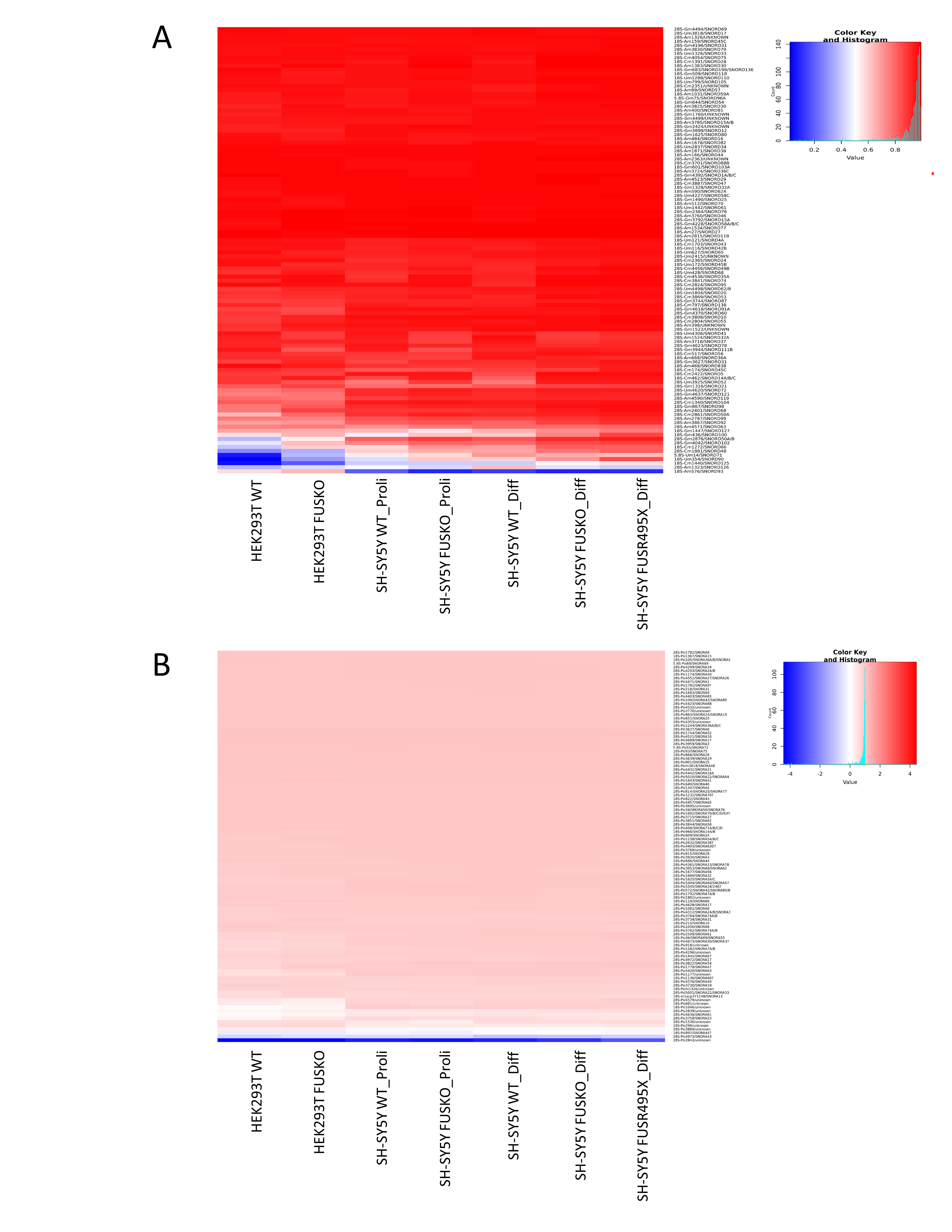
**

**Supplementary Fig. S3.** Heatmaps presenting 2’-O-Me (A) and pseudouridine (B) levels in all cells analyzed (HEK293T WT and FUS KO, SH-SY5Y proliferating (Proli) WT and FUS KO, SH-SY5Y differentiated (Diff) WT, FUS KO and FUS R495X), representing the mean of three biological replicates from each cell type. Heatmaps were created using gplots 3.1.3 R package (https://CRAN.R-project.org/package=gplots). The blue and red colors show a low and high proportion of modification, respectively.


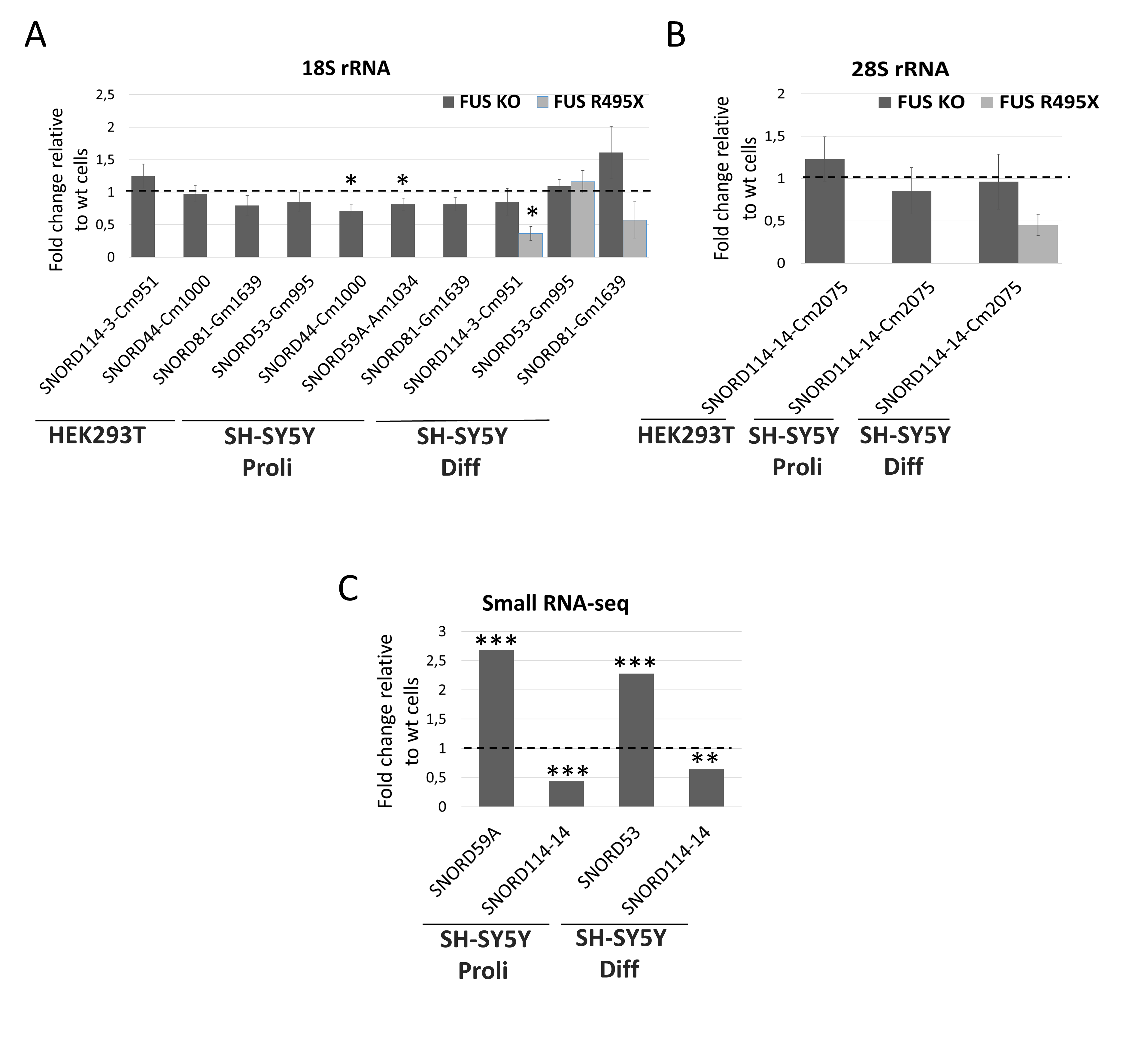


**Supplementary Fig. S4.** RT-qPCR analysis of relative expression level of selected C/D box snoRNAs predicted to guide methylation at putative new positions in 18S rRNA (A) and 28S rRNA (B). Small RNA-seq results of relative expression levels of selected snoRNAs that guide methylation and pseudouridylation of residues in 18S rRNA and 28S rRNA in SH-SY5Y proliferating and differentiated cells (C). Bars represent the mean and error bars indicate the SD of three biological replicates. HEK293T FUS KO cells, proliferating (Proli) SH-SY5Y FUS KO cells, differentiated (Diff) SH-SY5Y FUS KO cells, and FUS R495X cells were compared to WT cells, respectively. P-values were calculated using Student’s t-test, and the statistical significance is defined as follows: *P ≤ 0.05; **P ≤ 0.01; ***P ≤ 0.001.


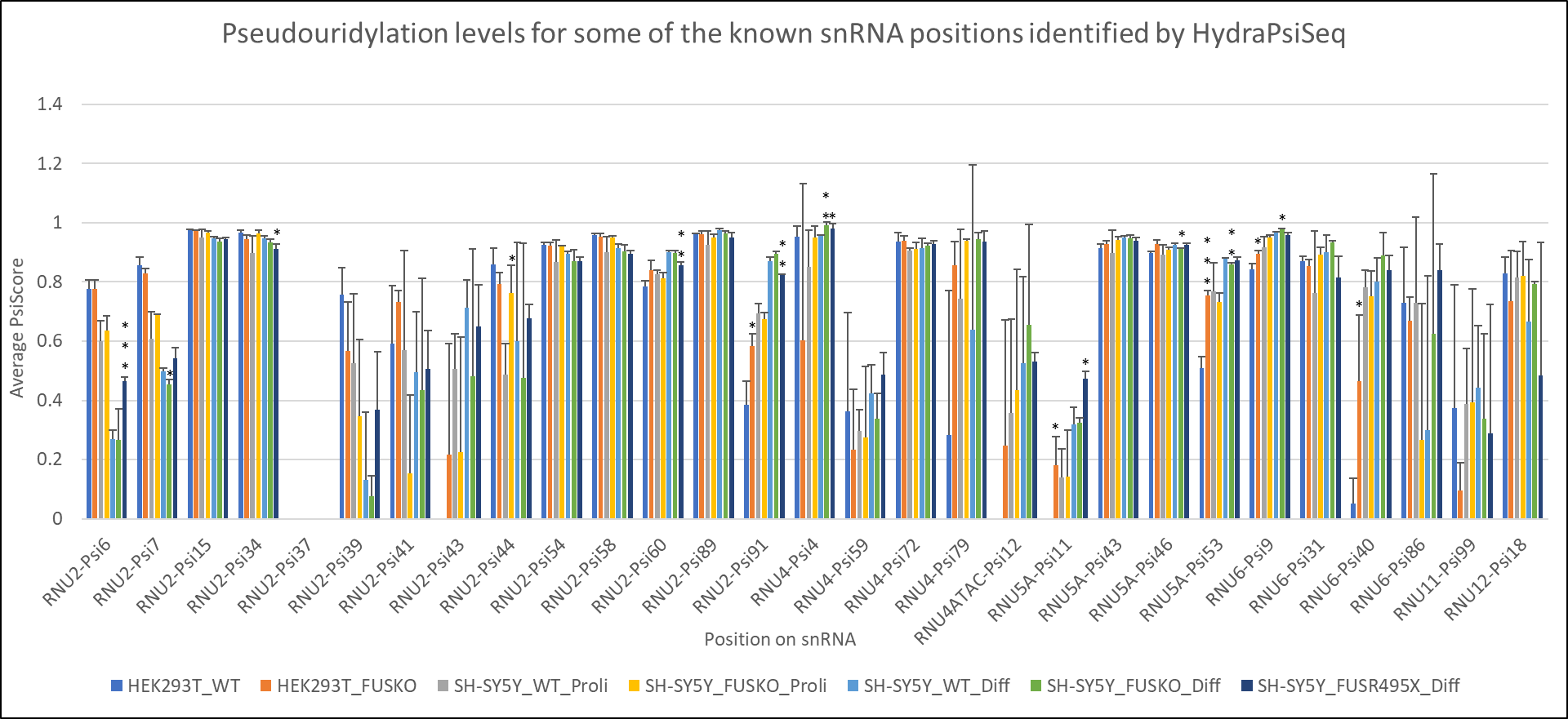


B


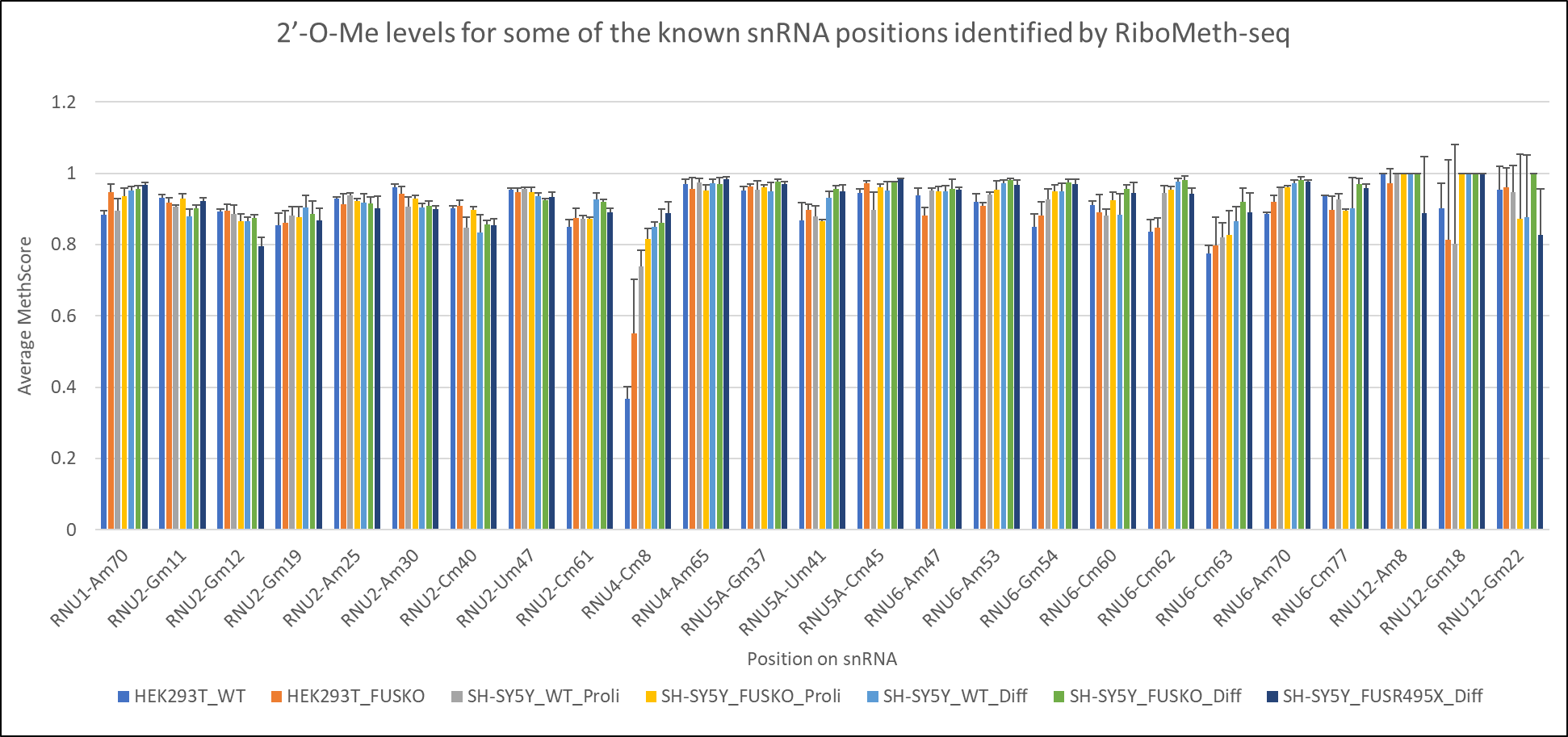


A

**Supplementary Fig. S5.** Distribution and extent of 2’-O-Me (A) and pseudouridylation (B) of residues in snRNAs. Bars represent the mean of MethScore and PsiScore and error bars indicate the SD between three biological replicates from each cell line. P-values were calculated using Student’s t-test, and the statistical significance is defined as follows: *P ≤ 0.05; **P ≤ 0.01; ***P ≤ 0.001.

**
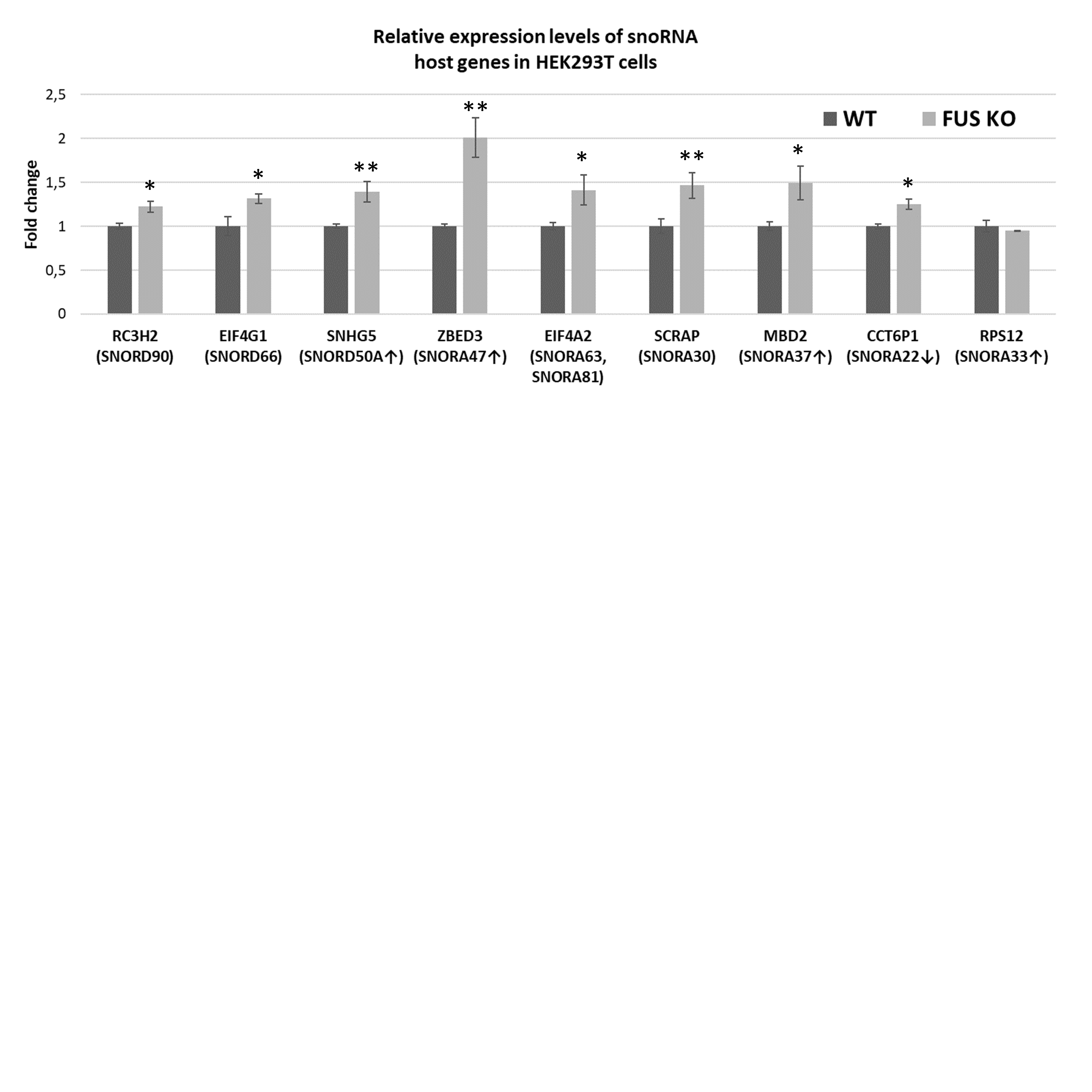
**

**Supplementary Fig. S6.** RT**-**qPCR analysis of relative expression levels of host genes of selected snoRNAs in HEK293T WT and FUS KO cells. Bars represent the mean and error bars indicate the SD of three biological replicates. P-values were calculated using Student’s t-test, and the statistical significance is defined as follows: *P ≤ 0.05; **P ≤ 0.01. Arrows next to snoRNA names indicate the direction of changes in the expression of hosted snoRNAs in FUS KO cells relative to WT cells.

**
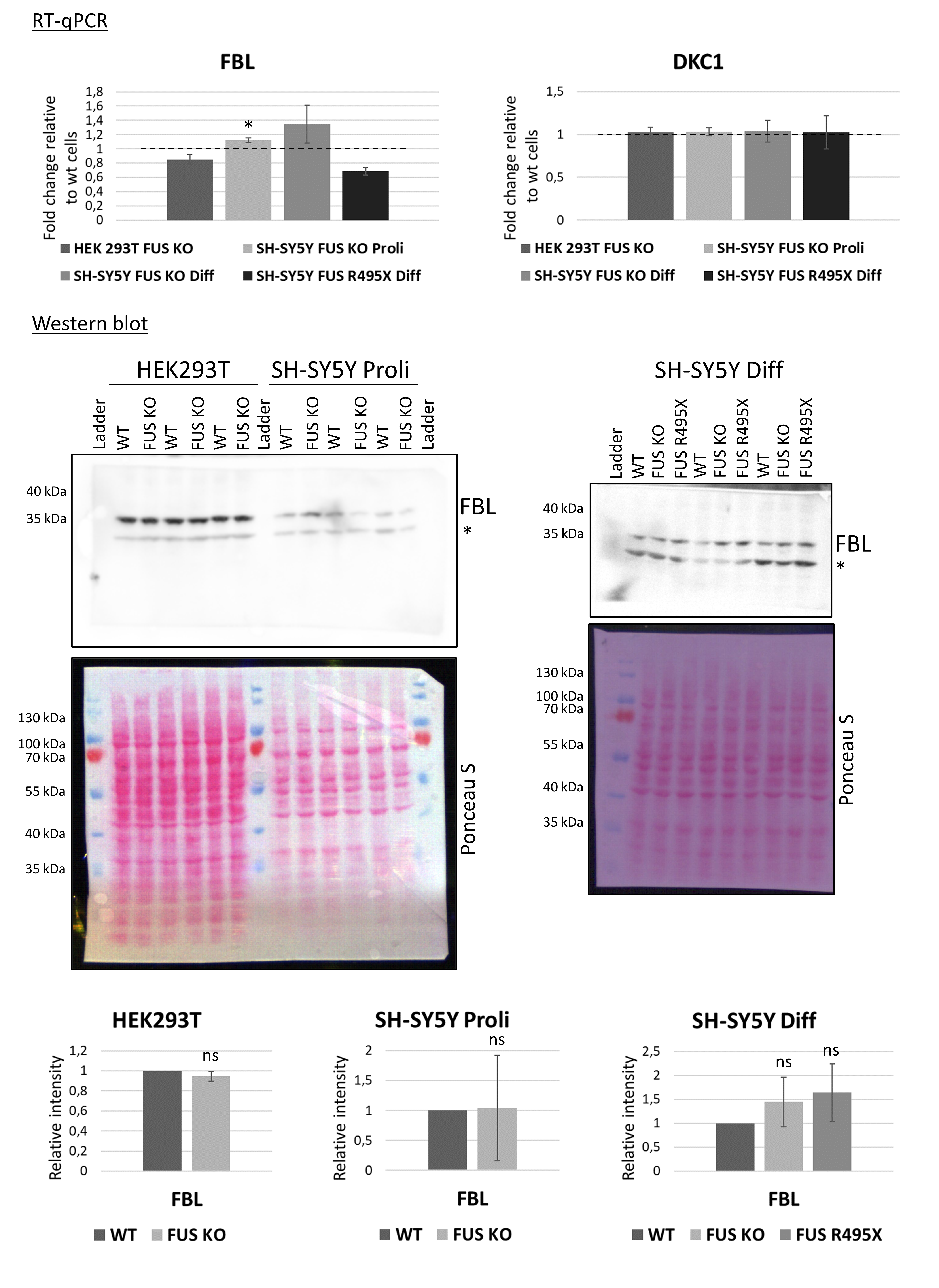
**

**
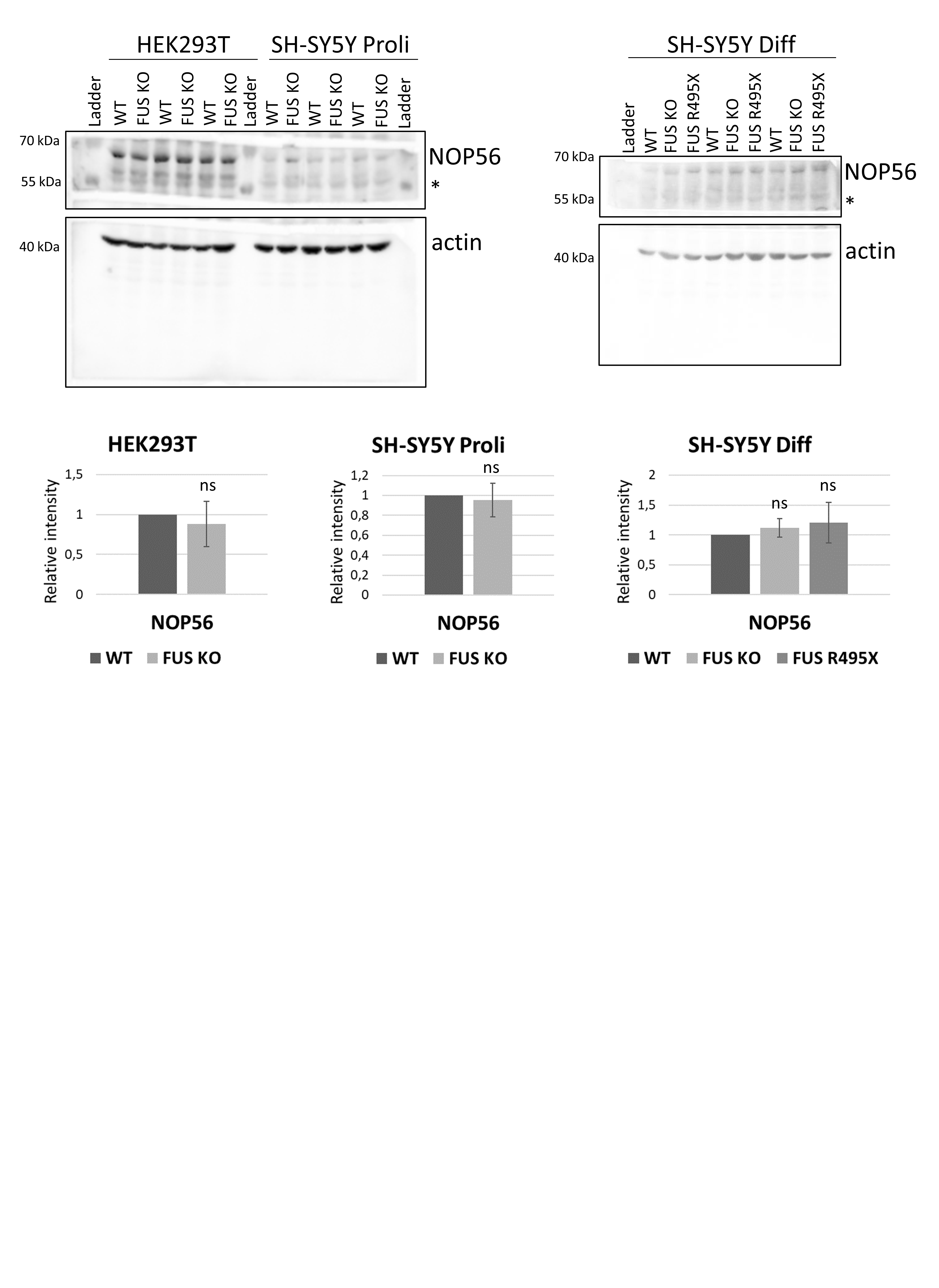
**

**Supplementary Fig. S7.** RT-qPCR and Western blot and immunodetection to analyze the expression of fibrillarin (FBL), NOP56, and dyskerin (DKC1) in FUS KO cells and FUS R495X cells in comparison to WT cells. For staining, anti-FIB, anti-NOP56 and anti-actin antibodies were used. Actin level or Ponceau S-stained membranes were used as loading controls for normalization in Western blot densitometrical analyses. Bars represent the mean and error bars indicate the SD of three biological replicates. P-values were calculated using Student's t-test, and the statistical significance is defined as follows: *P ≤ 0.05. *: unspecific signal. Original images of full-length blots are not provided, because blots were cut prior to incubation with appropriate antibodies.


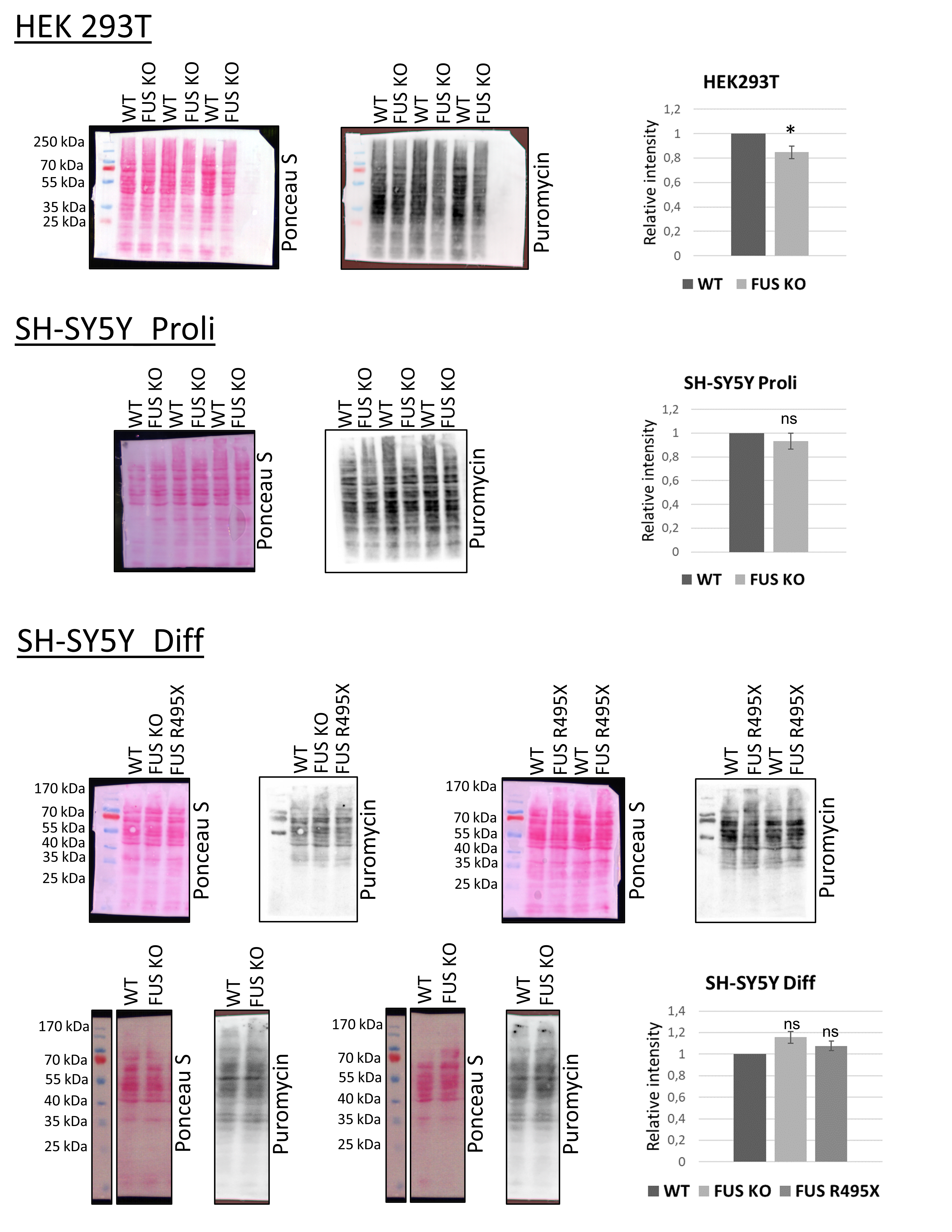


**Supplementary Fig. S8.** SUnSET assay to analyze global translation efficiency in all cells analyzed (HEK293T WT and FUS KO, SH-SY5Y proliferating (Proli) WT and FUS KO, SH-SY5Y differentiated (Diff) WT, FUS KO and FUS R495X). Ponceau S-stained membranes were used as loading controls. The graphs represent the mean of three biological replicates and were performed using the intensity of puromycinylated proteins normalized to Ponceau S signal in each lane. Error bars indicate the SD of three biological replicates. P-values were calculated using Student’s t-test, and the statistical significance is defined as follows: *P ≤ 0.05.


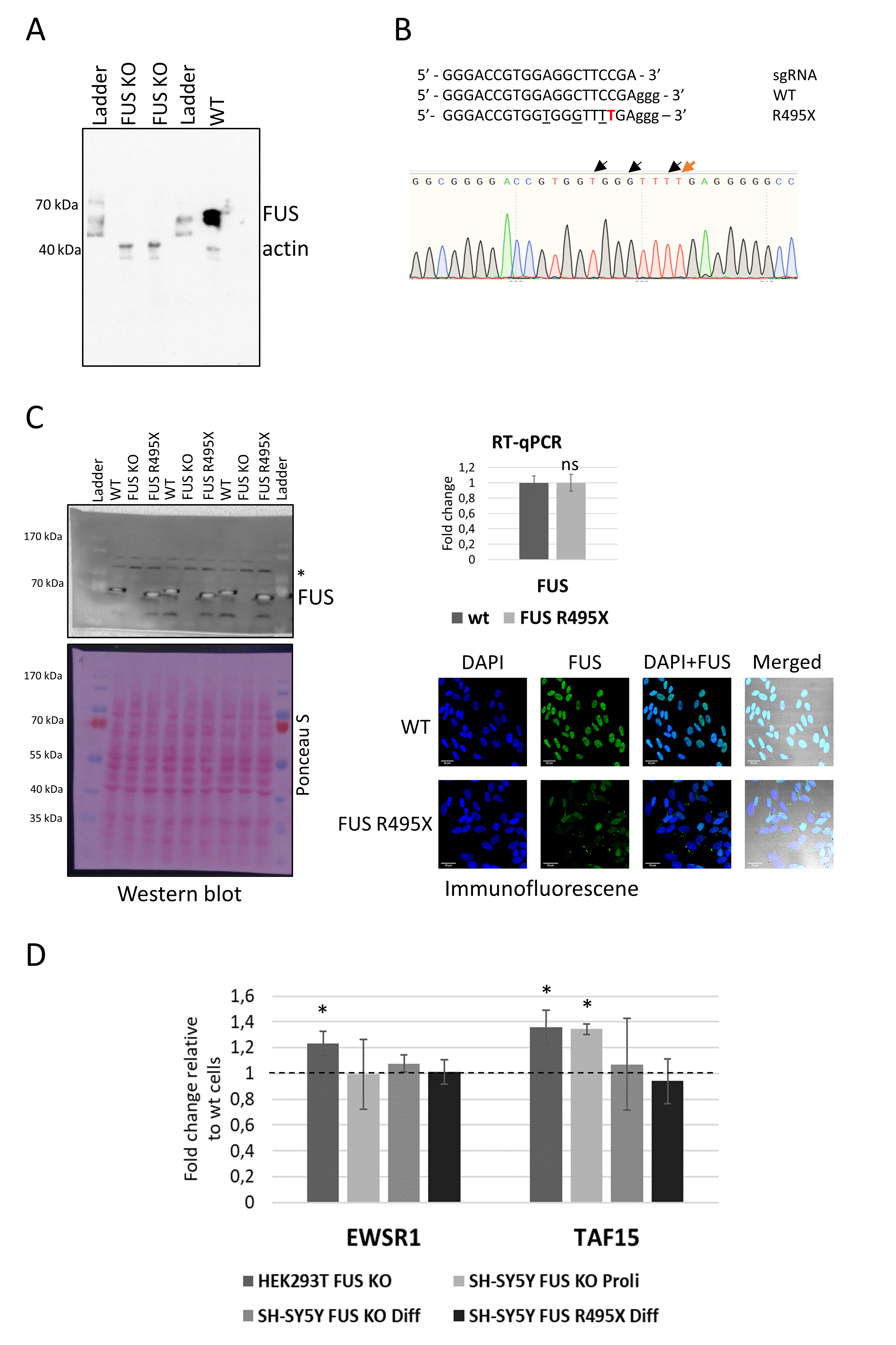


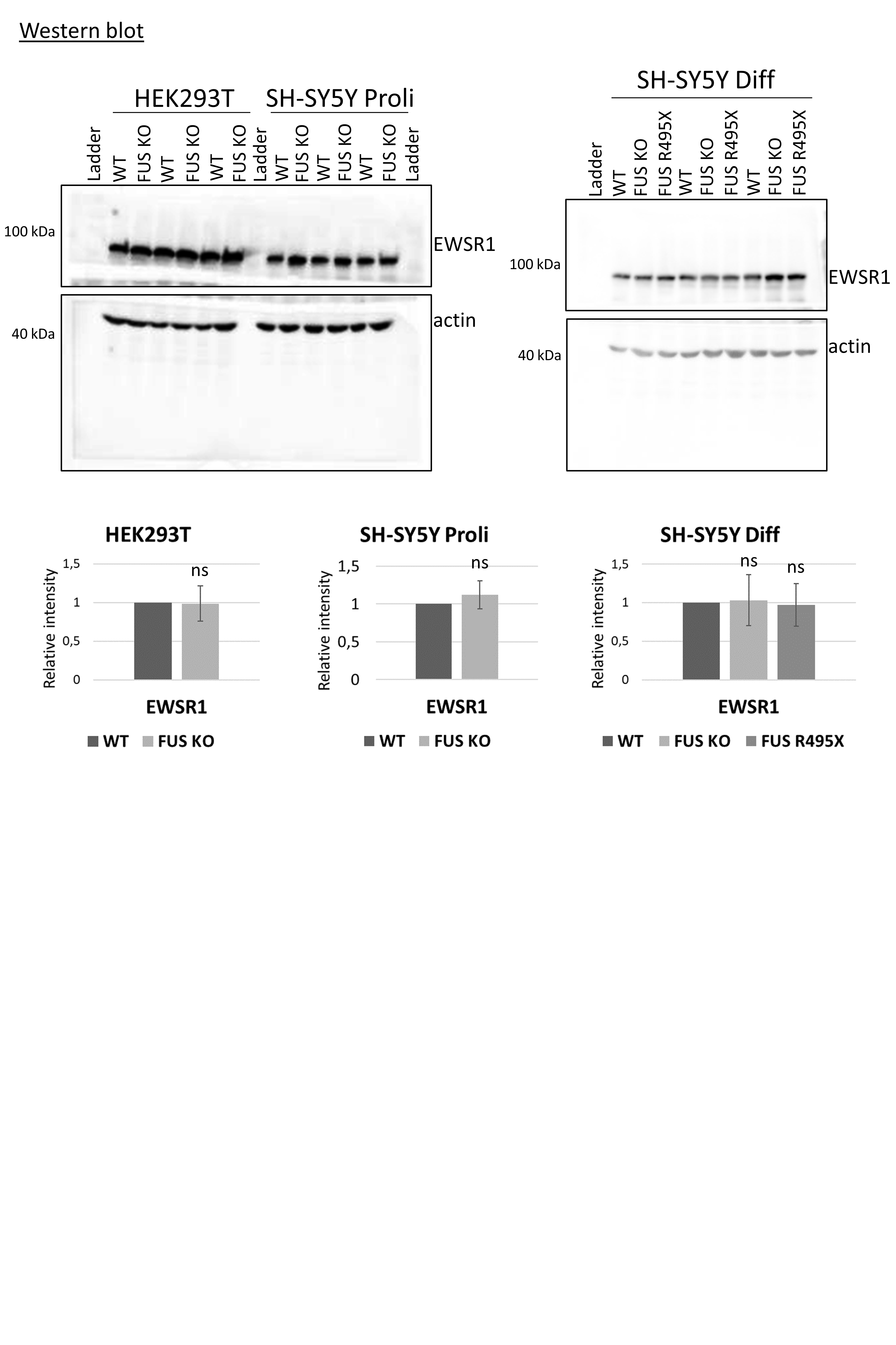


**Supplementary Fig. S9.** Western blot and immunodetection to confirm the knockout of *FUS* gene in HEK293T cells. For staining, anti-FUS and anti-actin antibodies were used. HEK293T FUS KO cells show no band corresponding to FUS protein (A). Representative sequence alignment and sequencing chromatogram of CRISPR/Cas9-induced FUS R495X mutation (arginine to stop codon, marked in red). Small letters correspond to PAM sequence. Silent mutations in sgRNA seeding regions are underlined (B). RT-qPCR, Western blot and immunodetection as well as immunofluorescence to confirm the expression and partial cytoplasmic mislocalization of FUS R495X in differentiated SH-SY5Y cells. Scale bar 20 µm (C). RT-qPCR and Western blot and immunodetection to test the expression level of TAF15 and EWSR1 in all cells analyzed (HEK293T WT and FUS KO, SH-SY5Y proliferating (Proli) WT and FUS KO, SH-SY5Y differentiated (Diff) WT, FUS KO and FUS R495X) (D). Actin level was used as loading control for normalization in Western blot densitometrical analyses. Bars represent the mean and error bars indicate the SD of three biological replicates. P-values were calculated using Student's t-test, and the statistical significance is defined as follows: *P ≤ 0.05. *: unspecific signal. Original images of full-length blots are not provided, because blots were cut prior to incubation with appropriate antibodies.

**Supplementary Table S3.** Representative results from Snoscan.

snoDB database fasta file was provided to the Snoscan tool with human ribosomal RNAs SSU and LSU as target RNAs. Snoscan was able to identify known 2’-O-Me sites, one example is mentioned here (28S-Cm2421). Alignment of putative new 2’-O-Me sites with guide snoRNAs predicted by the Snoscan tool are mentioned below:

**1. 18S-Cm951 – Putative guide C/D box snoRNA SNORD114-3/12/14**

>> SNORD114-3 13.25 (10-76) Cmpl: NU_167214.1:109078-110946-Cm951 (-) 12/1 bp Gs-DpBox: 20 (11) Len: 67 TS

No known meth site found Guide Seq Sc: -4.33 (15.04 -2.68 -15.69 -1.00)

*

Db seq: 5'- CGCCGCUAGAGGU -3' NU_167214.1:109078-110946 (948-956)

||||:|:|| |||

Qry seq: 3'- AGUUUGCGGUGGUCACCA -5' SNORD114-3 (32-20)

Strong terminal stem: +-[C Box] -N- CCAGGU-D - 5' Stem Sc: 5.76 (6 bp)

| ||||||

+---[D Box] - GGUCCAA - 3' Stem Transit Sc: -0.63

>Summary [ C Box ] -- -- [ Cmpl/ Mism ] X [D'Bx] -- -- [D Bx] Length

>Meth Cm 951 [AUGAUGA] -- 0 bp -- [ 12 / 1 ] 1 [UUGA] -- 33 bp -- [CUGA] 67 bp

>Sc 13.25 [ 11.42 ] -- -6.55 -- [ 15.04 bits ] [3.67] -3.54 [8.05]

Candidate sequence:
>SNORD114-3 13.25 (10-76) Cmpl: NU_167214.1:109078-110946-Cm951 Len: 67

ACCAATGATGACCACTGGTGGCGTTTGAGTCATGGACGATGAATACTACGTGTCTGAAACTCTGAGG

**2. 18S-Gm995 – Putative guide C/D box snoRNA SNORD53**

>> SNORD53 15.45 (7-81) Cmpl: NU_167214.1:109078-110946-Gm995 (-) 12/2 bp Gs-DpBox: 22 (16) Len: 75 TS

No known meth site found Guide Seq Sc: -1.19 (18.18 -2.68 -15.69 -1.00)

*

Db seq: 5'- AGCGAAAGCAUUUG -3' NU_167214.1:109078-110946 (992-1005)

||||||| ||| ||

Qry seq: 3'- GGUCGUCGCUUUGGUAUAC -5' SNORD53 (35-22)

Terminal stem: +-[C Box] -N- CGUADRON - 5' Stem Sc: 2.31 (4 bp)

| ||||

+---[D Box] - GCAU - 3' Stem Transit Sc: -0.63

>Summary [ C Box ] -- -- [ Cmpl/ Mism ] X [D'Bx] -- -- [D Bx] Length

>Meth Gm 995 [AUGAUGA] -- 4 bp -- [ 12 / 2 ] 1 [CUGG] -- 35 bp -- [CUGA] 75 bp

>Sc 15.45 [ 11.42 ] -- -3.32 -- [ 18.18 bits ] [2.94] -3.54 [8.05]

Candidate sequence:
>SNORD53 15.45 (7-81) Cmpl: NU_167214.1:109078-110946-Gm995 Len: 75

TGCTATGATGACATCCATATGGTTTCGCTGCTGGCTGAGTTTCAGAGATGACACCTTTCTCTTGGCTGTCTGAGC

**3. 18S-Cm1000 - Putative guide C/D box snoRNA SNORD44**

>> SNORD44 12.92 (2-61) Cmpl: Hu-18S-Cm1000 (-) 10/2 bp Gs-DpBox: 16 (15) Len: 60

No known meth site found Guide Seq Sc: -5.42 (14.60 -1.98 -15.69 -2.35)

*

Db seq: 5'- GAAAGCAUUUGC -3' Hu-18S (995-1006)

| |||||||||

Qry seq: 3'- AGUCAGUCGUAAACG -5' SNORD44 (27-16)

No terminal stem: +-[C Box] -N- GUCC - 5' Stem Sc: -1.71 (1 bp)

| |

+---[D Box] - CU - 3' Stem Transit Sc: -1.50

>Summary [ C Box ] -- -- [ Cmpl/ Mism ] X [D'Bx] -- -- [D Bx] Length

>Meth Cm1000 [AUGAUGA] -- 3 bp -- [ 10 / 2 ] -1 [CUGA] -- 25 bp -- [CUGA] 60 bp

>Sc 12.92 [ 11.42 ] -- -3.32 -- [ 14.60 bits ] [7.09] -2.80 [8.05]

Candidate sequence:

>SNORD44 12.92 (2-61) Cmpl: Hu-18S-Cm1000 Len: 60

CTGGATGATGATAAGCAAATGCTGACTGAACATGAAGGTCTTAATTAGCTCTAACTGACT

**4. 18S-Am1034 - Putative guide C/D box snoRNA SNORD59A**

>> SNORD59A 12.91 (3-72) Cmpl: Hu-18S-Am1034 (-) 10/0 bp Gs-DpBox: 26 (24) Len: 70 TS

No known meth site found Guide Seq Sc: -0.55 (18.91 -2.06 -15.69 -1.72)

*

Db seq: 5'- AACGAAAGUC -3' Hu-18S (1030-1042)

||||||||||

Qry seq: 3'- CUUCUUGCUUUCAG -5' SNORD59A (35-26)

Terminal stem: +-[C Box] -N- CUUCC - 5' Stem Sc: 4.29 (5 bp)

| |||||

+---[D Box] - GAAGG - 3' Stem Transit Sc: -0.63

>Summary [ C Box ] -- -- [ Cmpl/ Mism ] X [D'Bx] -- -- [D Bx] Length

>Meth Am1034 [AUGAUGA] -- 12 bp -- [ 10 / 0 ] 0 [CUUC] -- 27 bp -- [CUGA] 70 bp

>Sc 12.91 [ 11.42 ] -- -3.97 -- [ 18.91 bits ] [-1.57] -3.54 [8.05]

Candidate sequence:
>SNORD59A 12.91 (3-72) Cmpl: Hu-18S-Am1034 Len: 70

TTCTATGATGATTTTATCAAAATGACTTTCGTTCTTCTGAGTTTGCTGAAGCCACATTTA

GGTACTGAGA

**5. 18S-Am1639 - Putative guide C/D box snoRNA SNORD81**

>> SNORD81 12.30 (5-72) Cmpl: Hu-18S-Gm1639 (-) 9/1 bp Gs-DpBox: 30 (26) Len: 68 TS

No known meth site found Guide Seq Sc: -7.33 (11.42 -2.06 -15.69 -1.00)

*

Db seq: 5'- GAGGGAAUUC -3' Hu-18S (1636-1646)

|||:|| |||

Qry seq: 3'- AGUCACUCUCUCAAG -5' SNORD81 (39-30)

Strong terminal stem: +-[C Box] -N- AUAAGAC - 5' Stem Sc: 6.14 (7 bp)

| |||||||

+---[D Box] - UAUUCUG - 3' Stem Transit Sc: -0.63

>Summary [ C Box ] -- -- [ Cmpl/ Mism ] X [D'Bx] -- -- [D Bx] Length

>Meth Gm1639 [AUGAUGA] -- 14 bp -- [ 9 / 1 ] 1 [CUGA] -- 22 bp -- [CUGA] 68 bp

>Sc 12.30 [ 11.42 ] -- -8.14 -- [ 11.42 bits ] [7.09] -3.69 [8.05]

Candidate sequence:

>SNORD81 12.30 (5-72) Cmpl: Hu-18S-Gm1639 Len: 68

ATACATGATGATCTCAATCCAACTTGAACTCTCTCACTGATTACTTGATGACAATAAAAT

ATCTGATA

**6. 28S-Cm2075 - Putative guide C/D box snoRNA SNORD114-14**

>> SNORD114-14 22.70 (10-76) Cmpl: NU_167214.1:113348-118417-Cm2084 (-) 10/0 bp Gs-DpBox: 23 (14) Len: 67 TS

No known meth site found Guide Seq Sc: 2.74 (21.49 -2.06 -15.69 -1.00)

*

Db seq: 5'- CGCCGGCAGU -3' NU_167214.1:113348-118417 (2081-2089)

||||||||||

Qry seq: 3'- AGUAUGCGGCCGUCA -5' SNORD114-14 (32-23)

Strong terminal stem: +-[C Box] -N- CCAGGU-D - 5' Stem Sc: 5.76 (6 bp)

| ||||||

+---[D Box] - GGUCCA - 3' Stem Transit Sc: -0.63

>Summary [ C Box ] -- -- [ Cmpl/ Mism ] X [D'Bx] -- -- [D Bx] Length

>Meth Cm2084 [AUGAUGA] -- 2 bp -- [ 10 / 0 ] 1 [AUGA] -- 33 bp -- [CUGA] 67 bp

>Sc 22.70 [ 11.42 ] -- -3.32 -- [ 21.49 bits ] [2.81] -3.54 [8.05]

Candidate sequence:

>SNORD114-14 22.70 (10-76) Cmpl: NU_167214.1:113348-118417-Cm2084 Len: 67

ACCAATGATGACAACTGCCGGCGTATGAGTGTTGGGTGATGAATAATACGTGTCTAGAACTCTGAGG

**7. 28S-Cm2421 – Guide C/D box snoRNA SNORD5**

>> SNORD5 19.01 (8-75) Cmpl: NU_167214.1:113348-118417-Cm2422 (-) 13/0 bp Gs-DpBox: 26 (19) Len: 68

No known meth site found Guide Seq Sc: 5.68 (25.05 -2.68 -15.69 -1.00)

*

Db seq: 5'- CAGCAGUUGAACA -3' NU_167214.1:113348-118417 (2419-2433)

|||||||||||||

Qry seq: 3'- AGUAAGUCGUCAACUUGU -5' SNORD5 (38-26)

Possible terminal stem: +-[C Box] -N- ACUUGDRO - 5' Stem Sc: -0.19 (3 bp)

| ||:

+---[D Box] - CAGAG - 3' Stem Transit Sc: -1.50

>Summary [ C Box ] -- -- [ Cmpl/ Mism ] X [D'Bx] -- -- [D Bx] Length

>Meth Cm2422 [AUGAUGA] -- 7 bp -- [ 13 / 0 ] 1 [AUGA] -- 26 bp -- [CUGA] 68 bp

>Sc 19.01 [ 11.42 ] -- -3.32 -- [ 25.05 bits ] [2.81] -3.54 [8.05]

Candidate sequence:
>SNORD5 19.01 (8-75) Cmpl: NU_167214.1:113348-118417-Cm2422 Len: 68

TCAGATGATGAATTTAACTGTTCAACTGCTGAATGATAACGGGCATGAACTAAAACTTAATTCTGAC

**Supplementary Table S5.** Primers used in PCR and RT-qPCR

| AGCTGCGGTTTCAGGTAGTC | FUS (Fwd) | |
| --- | --- | --- |
| ACTTTTAATGGGAACCAGAGGT | FUS (Rev) | |
| GTCTAATGATGAATTTCATAGGGCA | SNORD90 (Fwd) | |
| GTCTTCAGATTCCACAGTAGGAG | SNORD90 (Rev) | |
| CTGATGACTTCCTGTTAGTGCC | SNORD66 (Fwd) | |
| TCCTCAGATCCTCAGTTCCATC | SNORD66 (Rev) | |
| ACATGATGACAACTGGCTCCC | SNORD100 (Fwd) | |
| GCTGTAATCAGAAGGGTGACAT | SNORD100 (Rev) | |
| TGGCCAAGGATGAGAACTCTA | SNORD93 (Fwd) | |
| GGCCTCAGGTAAATCCTTTAATCC | SNORD93 (Rev) | |
| AGCCCCTCCTGATGATTC | SNORD125 (Fwd) | |
| TTCAGTCAACTTCTTAGAGGCTC | SNORD125 (Rev) | |
| TGTGATGATCTTATCCCGAACCT | SNORD50A (Fwd) | |
| ATCTCAGAAGCCAGATCCGT | SNORD50A (Rev) | |
| AGCTTAATGATGACTGTTTTTTTTGATTGCTTGA | SNORD102 (Fwd) | |
| AGCTTTCAGAGCCGGTGAAATGTGTTTTTC | SNORD102 (Rev) | |
| CATGATGAAATGCATGTTAAGTCCGT | SNORD126 (Fwd) | |
| GCTCAGAGCATGTGTTTAATCAGGC | SNORD126 (Rev) | |
| GATGATGACCCCAGGTAACTCTTG | SNORD48 (Fwd) | |
| GTCAGAGCGCTGCGGTGAT | SNORD48 (Rev) | |
| TGGTGCTGTGATGATGCCTTA | SNORD92 (Fwd) | |
| GCTCAGACACAGCCAAGGAA | SNORD92 (Rev) | |
| CAATGATGACCACTGGTGGCG | SNORD114-3 (Fwd) | |
| TTGGACCTCAGAGTTTCAGACA | SNORD114-3 (Rev) | |
| CCTGGATGATGATAAGCAAATGCTGACT | SNORD44 (Fwd) | |
| AGTCAGTTAGAGCTAATTAAGACCTTCATG | SNORD44 (Rev) | |
| TGATGACATCCATATGGTTTCGCTG | SNORD53 (Fwd) | |
| GCTCAGACAGCCAAGAGAAAG | SNORD53 (Rev) | |
| CCTTCTATGATGATTTTATCAAAATGACTTTCGTT | SNORD59A (Fwd) | |
| CCTTCTCAGTACCTAAATGTGGCTTCA | SNORD59A (Rev) | |
| CAGAATACATGATGATCTCAATCCAACTTGAAC | SNORD81 (Fwd) | |
| CAGAATATCAGATATTTTATTGTCATCAAGTAATCAGTG | SNORD81 (Rev) | |
| GACCAATGATGACAACTGCC | SNORD114-14 (Fwd) | |
| GACCTCAGAGTTCTAGACACGTATT | SNORD114-14 (Rev) | |
| GTGCTGTGTTGTCGTTCCCC | SNORA63 (Fwd) | |
| GCTGCTACAGGAGAATAGCAGA | SNORA63 (Rev) | |
| CACTTTCACAGTTCCTTCCCC | SNORA30 (Fwd) | |
| TCAAGGGTTTTCTCTCAGCACC | SNORA30 (Rev) | |
| AGCACTTTCACAGGTCCTCCC | SNORA37 (Fwd) | |
| GGCAAGGATGCCAACAAAGGT | SNORA37 (Rev) | |
| TTGCACAGTGAACACCCAAGT | SNORA22 (Fwd) | |
| CAGAGGAGAAGAGCAGGCAAT | SNORA22 (Rev) | |
| AGCCAGCCAATGAATCTGCTT | SNORA33 (Fwd) | |
| AGGCTCGTAACATGGCTTTACT | SNORA33 (Rev) | |
| CCTTCCACCGGTTAAGACCTC | SNORA47 (Fwd) | |
| CAAATGTCGGCCAGCACAGC | SNORA47 (Rev) | |
| TTCGTAACCCGTTAGCCTGG | SNORA54 (Fwd) | |
| AGTCAGTCATGTGTCGCTGG | SNORA54 (Rev) | |
| GCTTCGGAAAGGGAGGGAAA | SNORA7A/7B (Fwd) | |
| CTGTCGCAGAGTGTCTTCCA | SNORA7A/7B (Rev) | |
| CTCCAACTGCATGCAAGAGC | SNORA44 (Fwd) | |
| ATAGGAAAGCTGAGTGGCAGC | SNORA44 (Rev) | |
| ATTGCAGACACTAGGACCATGT | SNORA81 (Fwd) | |
| AGAAAGAGGTCCACCCCAGT | SNORA81 (Rev) | |
| GTTGGCACCACAGACAGTTG | SNORA43 (Fwd) | |
| AAACCATTCTCAGTGCCCAC | SNORA43 (Rev) | |
| GGTGGAGGAAGAAGGTCGTG | RC3H2 (For) |  |
| CTGACAGCGGCCCATAGATT | RC3H2 (Rev) |  |
| ACCTGAGGAACTGCTCAACG | EIF4G1 (For) |  |
| AAGGAGCCGTAGCTGGAGTA | EIF4G1 (Rev) |  |
| GCTGGAGGTGTAATGGACG | RPS12 (For) |  |
| TTCGCGAATTCCACGTGCT | RPS12 (Rev) |  |
| CTTAATTGGGGCGGAGGGTT | SNHG5 (For) |  |
| ATCCGAATTGCACACAACGC | SNHG5 (Rev) |  |
| AGAGCGGGAAGAGGATGGAT | MBD2 (For) |  |
| TCGCTCTTGCCAGCACTTAG | MBD2 (Rev) |  |
| GAATGCAGCGCTGTGTCTTT | ZBED3 (For) |  |
| CCGAGATGGTAGATCCCCCT | ZBED3 (Rev) |  |
| GCTGGCGTTCAACATTAGCG | CCT6P1 (For) |  |
| ATCTTTATGGTCCCTTTGGGC | CCT6P1 (Rev) |  |
| GCTCTGACTGTGAACCAGAGG | SRCAP (For) |  |
| TGACTGACTGCTACTATCCTCC | SRCAP (Rev) |  |
| AGCAACTGGAATGAGATTGTTGA | EIF4A2 (For) |  |
| CTGCTGAATAGCGGAAGGCT | EIF4A2 (Rev) |  |
| CTGTAGTGCGCTATGCCGAT | 7SL RNA (For) |  |
| CACGGGAGTTTTGACCTGCT | 7SL RNA (Rev) |  |
| ACTCCAGTTATGGACAAAGTCAGT | TAF15 (For) |  |
| TGGCTGGTCATAGGAAGGTG | TAF15 (Rev) |  |
| ATGGCGTCCACGGATTACAG | EWSR1 (For) |  |
| CCATATGCCTGGGTGGTCTG | EWSR1 (Rev) |  |
| GGCGGATGCGGAAGTAAT | DKC1 (For) |  |
| CCACTGAGACGTGTCCAACTT | DKC1 (Rev) |  |
| GAGGCTTCCATTCTGGTGGCAA | FBL (For) |  |
| CAGGTTCTTGGTGACCAGTGCA | FBL (Rev) |  |
| CAGTGTTACAGCTCTTTTAGAATTTG | U7 snRNA (For) |  |
| TTCCGGTAAAAAGCCAGAAA | U7 snRNA (Rev) |  |
| GATACCATGATCACGAAGGTGGTT | U1 snRNA (For) |  |
| CACAAATTATGCAGTCGAGTTTCC | U1 snRNA (Rev) |  |
| CTCAACGACCACTTTGTCAAGCT | GAPDH (For) |  |
| TCTTACTCCTTGGAGGCCATGT | GAPDH (Rev) |  |
